# Perspectives and Performance of First-Year Medical and Dental Students in Anatomy Education Through Cadaveric Dissection: A Cross-Sectional Study

**DOI:** 10.7759/cureus.78648

**Published:** 2025-02-06

**Authors:** Archisha Bansal, Anupam Gajrani, Puneet Sharma, Deepak Kumar, Sanchita Gulati, Shweta Bansal, Gaurav Bansal, Seema Gupta

**Affiliations:** 1 Department of Anatomy, School of Medical Sciences and Research, Greater Noida, IND; 2 Department of Anatomy, Rajshree Medical Research Institute, Bareilly, IND; 3 Department of Periodontics, Jan Nayak Chaudhary Devi Lal Dental College, Sirsa, IND; 4 Department of Pedodontics and Preventive Dentistry, Jan Nayak Chaudhary Devi Lal Dental College, Sirsa, IND; 5 Department of Conservative Dentistry and Endodontics, Jan Nayak Chaudhary Devi Lal Dental College, Sirsa, IND; 6 Department of Periodontics, Kothiwal Dental College and Research Centre, Moradabad, IND; 7 Department of Neurosurgery, Fortis Hospital, Noida, IND; 8 Department of Orthodontics, Kothiwal Dental College and Research Centre, Moradabad, IND

**Keywords:** cadavers, dental, dissection, medical, questionnaire, survey

## Abstract

Introduction: Anatomical education serves as the cornerstone of medical and dental training, and cadaveric dissection is traditionally regarded as the gold standard for teaching human anatomy. This study explored the attitudes and perceptions of first-year Bachelor of Medicine and Bachelor of Surgery (MBBS) and Bachelor of Dental Surgery (BDS) students toward cadaveric dissection and its impact on their academic performance and professional development.

Materials and methods: A cross-sectional survey was conducted in the Department of Anatomy. A structured questionnaire comprising 10 questions assessed the students' attitudes, perceptions, and preferences toward cadaveric dissection. The sample included 500 first-year MBBS and BDS students from four institutions. Data were collected anonymously using Google Forms and analyzed. Statistical tests, including chi-square and independent t-tests, were used to compare the responses and formative scores between the groups.

Results: A total of 450 medical and dental students responded, with 162 individuals (36.00%) participating in the BDS curriculum focused on anatomical studies, whereas 288 individuals (64.00%) were engaged in the MBBS program, resulting in an impressive response rate of 90%. Among medical students, 105 (36.5%%) were males, and 183 (63.5%) were females. Among dental students, 66 (40.7%) were males, and 96 (59.3%) were females. Dental students exhibited greater empathy and appreciation for cadaveric dissection (p=0.001), potentially related to the practical skills required in dentistry. However, medical students scored significantly higher on cadaveric dissection-based assessments (p<0.001), reflecting their broader and more detailed anatomical curricula. Despite these differences, students across both groups emphasized the importance of cadaveric dissection in developing clinical and professional skills.

Conclusion: Cadaveric dissection remains an indispensable tool in anatomy education, fostering a deep understanding of human anatomy and instilling empathy and professionalism. For perception, dental students significantly favored cadaveric dissection for providing a better learning experience than alternative methods, improving spatial understanding, and aiding clinical correlation understanding. Regarding attitude, dental students more strongly associated cadaveric dissection with respect and dignity, emotional comfort, and influencing attitudes toward life and death. In practice, dental students showed greater agreement that cadaveric dissection should remain integral to education and benefit their future careers.

## Introduction

Historically, the study of anatomy has been acknowledged as a fundamental component of medical education, providing essential groundwork for comprehending the human body and its intricacies [1]. Conventionally, the dissection of cadavers has been regarded as the premier method in anatomical education, delivering an unmatched experiential learning opportunity that enables medical students to investigate human anatomy within a 3D framework [1]. This approach not only augments students' understanding of anatomical entities but also cultivates vital competencies, including collaboration, professional conduct, and ethical awareness [2]. The hands-on and visual learning opportunities afforded by cadaveric dissection are fundamentally embedded in the medical educational framework, rendering it an essential component of medical training globally [2]. A study conducted by specialists from Austria, Brazil, Colombia, India, New Zealand, Nigeria, Spain, South Africa, the USA, and Uruguay substantiated the existence of cadaveric dissection in various manifestations and modalities within undergraduate educational programs at their respective institutions [3].

In contemporary times, the domain of medical education has experienced considerable evolution propelled by technological progress [4]. Tools for virtual dissection, including virtual reality, augmented reality, and 3D modeling, have surfaced as cutting-edge approaches to enhance conventional anatomy instruction [4]. A comprehensive systematic review conducted by Tene et al. [4] evaluated 28 studies that examined the efficacy of virtual reality and augmented reality in medical education. The findings indicated that although immersive virtual reality and augmented reality technologies show significant potential in improving medical training, further extensive research is necessary to ascertain their conclusive effectiveness. Virtual tools additionally offer adaptability, access, and the potential for iterative practice devoid of the limitations linked to cadaveric dissection, including restricted cadaver availability, ethical dilemmas, and possible psychological unease among learners [5].

Notwithstanding these technological advancements, the discourse regarding the most efficacious approach to teaching anatomy persists in a state of ambiguity [6]. Advocates of cadaveric dissection contend that it provides an unparalleled and indispensable opportunity for learners to cultivate practical competencies and deep comprehension of human anatomy, elements that are challenging to emulate through virtual modalities [7]. Ajani and Oladapo [8] evaluated the perceptions of medical and dental students concerning the utility of cadaveric dissection in education and determined that approximately 96.4% of respondents regarded cadaveric dissection as indispensable for mastering anatomical knowledge; concurrently, 55.6% expressed an interest in the subject, and 80.1% felt that it plays a substantial role in their prospective professional careers. Nevertheless, there is a paucity of research on this subject. Therefore, the present study was conducted to assess the perceptions, attitudes, and practices of first-year medical and dental students toward cadaveric dissection. The secondary objectives were to compare the differences in perception, attitude, and practice regarding cadaveric dissection between MBBS and BDS students and also to compare their performances in pre-university exams. By exploring students' preferences, confidence levels, and skill development associated with cadaveric dissection, this study aimed to provide insights into the effectiveness of this approach in contemporary medical or dental education.

## Materials and methods

Study design

This cross-sectional study was conducted in the Department of Anatomy, School of Medical Sciences and Research, Greater Noida, India, from July 2024 to August 2024 to investigate undergraduate medical students’ perceptions and preferences regarding cadaveric dissection as an education method. The need for ethical approval for the study was waived by the Institutional Ethics Committee (IEC) of the college. This study adhered to the principles of the Declaration of Helsinki. Informed consent was obtained from all the participants. The collected data were anonymized and stored securely to ensure confidentiality.

Sampling methodology

The convenience method of sampling was adopted, and the sample size was calculated using Calculator.net based on the expected proportion of students perceiving cadaver learning positively. Assuming a 30% proportion (maximum variability), a 95% confidence level, and a margin of error of ±5%, the sample size was determined using the following formula: N=Z^2^.P(1-P)/e^2^, where Z=1.96 (for a 95% confidence level), P=0.30 (expected proportion), and e=0.05 (margin of error). The calculation yielded the minimum required sample size of 399 students. To account for potential non-responses, the questionnaire was sent to all first-year Bachelor of Medicine and Bachelor of Surgery (MBBS), and Bachelor of Dental Surgery (BDS) students at four colleges (two medical and two dental colleges), ensuring adequate representation. The inclusion criteria were as follows: medical and dental students who have recently passed as first-year undergraduate students, received their pre-university marks, and are willing to participate in the study voluntarily. Students who had not yet begun their anatomy curriculum or were unwilling to complete the survey were excluded from this study.

Data collection tools

A structured questionnaire was designed to collect the data. Prior to its extensive application, the instrument underwent a comprehensive review and was piloted with a sample of 20 students to assess its clarity, reliability, and validity. Informed by feedback from the pilot, requisite modifications were implemented. The questionnaire was pretested and face-validated by subject matter experts to assess the attitudes, perceptions, and practices of the students regarding the various dissection methodologies employed in this study. The reliability, specifically the internal consistency, of the finalized questionnaire after the completion of the study was evaluated using Cronbach’s alpha, yielding a coefficient of 0.86. The questionnaire comprised three sections: section one consisted of the demographic details of the participants without disclosing their identity. The second section comprised 10 questions, of which the first five questions assessed perception (A1-A5), three questions (A6-A8) assessed attitude, and two questions (A9-A10) assessed practice on a four-point Likert scale (1=strong disagree, 4=strong agree).

Data collection procedure

**Figure 1:**
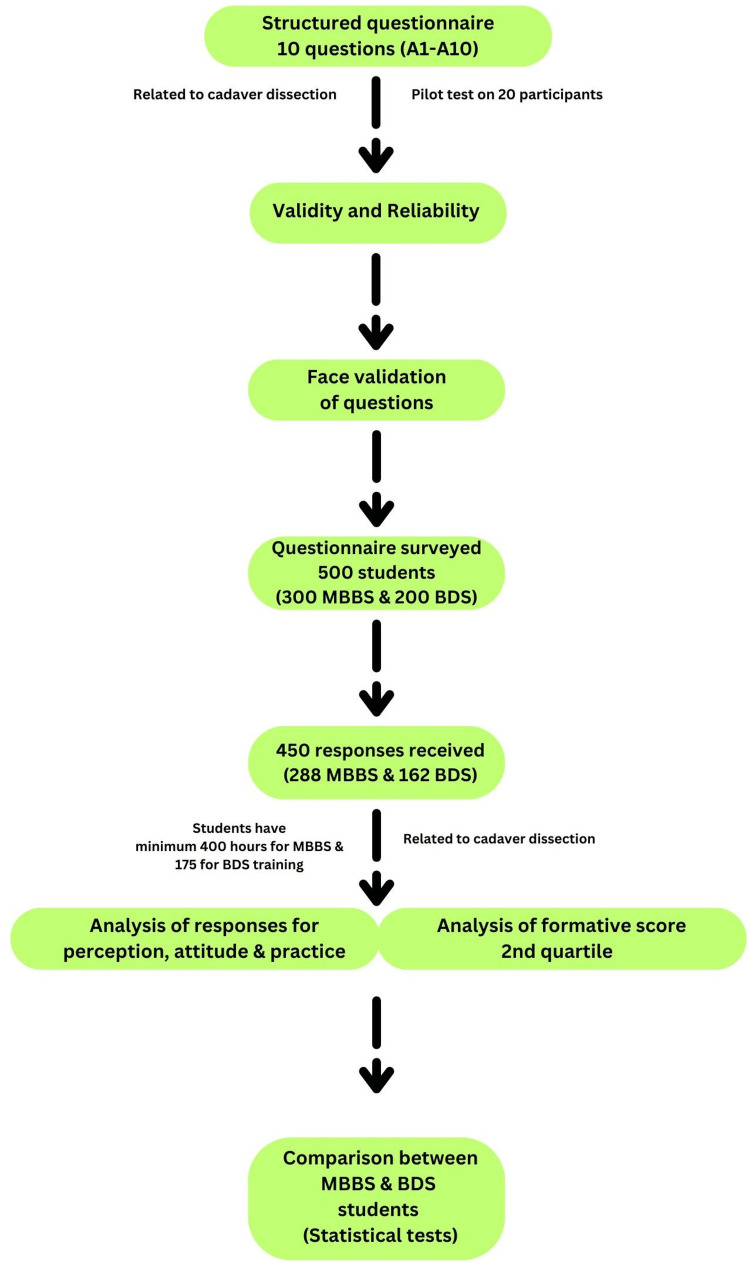
Flowchart for study design. MBBS: Bachelor of Medicine and Bachelor of Surgery, BDS: Bachelor of Dental Surgery

A total of 500 participants were administered a questionnaire after their pre-university examinations at two dental colleges and two medical colleges via a Google Form link, which was disseminated to all participants through WhatsApp, with instructions to complete the questionnaire within a duration of 20 minutes. The students were informed about the study objectives and assured of anonymity and confidentiality in handling their responses. The questionnaire emphasized voluntary participation and allowed students to opt-out at any point. Consent was obtained through questionnaire submission. Formative pre-university assessments conducted as part of the regular curriculum for students in the Department of Anatomy during the academic year were considered in order to determine their academic performance. The duration of cadaveric dissection was 175 hours for dental and 400 hours for medical students. The study design is shown in Figure 1.

Statistical analysis

Data were systematically entered into Microsoft Excel and analyzed using SPSS software (IBM Corp. Released 2013. IBM SPSS Statistics for Windows, Version 23.0. Armonk, NY: IBM Corp.). The data were checked for normality using the Shapiro-Wilk test, and the normal distribution of data was confirmed by a Q-Q plot. Categorical responses were presented as frequency and percentage, and the chi-square test was used to determine the association between responses. The mean and standard deviation (SD) for the second quartile of the formative score of participants were taken in the study groups. An independent Student’s t-test was used to compare formative scores between groups. The level of significance was set at p<0.05.

## Results

A cohort of 450 first-year students enrolled in medical and dental programs responded to the survey, with 162 (36.00%) BDS participants and 288 (64.00%) MBBS participants, resulting in a response rate of 90%. Among MBBS students, 105 (36.5%%) were males, and 183 (63.5%) were females. Among BDS students, 66 (40.7%) were males, and 96 (59.3%) were females. The age distribution of the participants showed that the majority were aged 20 or 21 years, with no statistically significant difference (p=0.150). Females comprised a higher percentage in both groups, and the difference in sex distribution between the groups was not statistically significant (p=0.562). Overall, demographic characteristics were comparable between the groups (Table 1).

**Table 1:** Age and sex distribution between groups. p-value>0.05: non-significant. Data is presented in the form of n (%). MBBS: Bachelor of Medicine and Bachelor of Surgery, BDS: Bachelor of Dental Surgery

Parameter	Groups	MBBS		BDS		p-value	Chi-square value
		288	%	162	%		
Age in years	19	48	16.7%	24	14.8%	0.150	5.307
	20	84	29.2%	66	40.7%		
	21	96	33.3%	54	33.3%		
	22	60	20.8%	18	11.1%		
Sex	Male	105	36.5%	66	40.7%	0.562	0.33
	Female	183	63.5%	96	59.3%		

The descriptive analysis of participant responses revealed significant differences in perceptions, attitudes, and practices regarding cadaveric dissection between the medical and dental students. Regarding perception, BDS students significantly favored cadaveric dissection for providing a better learning experience than alternative methods, improving spatial understanding, and aiding clinical correlation understanding. Regarding attitude, BDS students more strongly associated cadaveric dissection with respect and dignity, emotional comfort, and attitudes toward life and death. In practice, BDS students showed greater agreement that cadaveric dissection should remain integral to education and benefit their future careers. These results highlight stronger positive responses among dental students than their medical counterparts (Table 2).

**Table 2:** Descriptive analysis of responses from the participants. Perception (A1-A5), attitude (A6-A8), and practice (A9 and A10). *p-value<0.05: significant. MBBS: Bachelor of Medicine and Bachelor of Surgery, BDS: Bachelor of Dental Surgery

S.no	Questions	Response	MBBS		BDS		p-value	Chi-square value
			n=288	64.0%	n=162	36.0%		
A1	The use of cadaver dissection is essential for understanding human anatomy.	Strong agree	93	32.3%	75	46.3%	0.236	4.24
		Agree	144	50.0%	66	40.7%		
		Disagree	36	12.5%	15	9.3%		
		Strong disagree	15	5.2%	6	3.7%		
A2	Cadaver dissection provides a better learning experience than alternative methods such as virtual or model-based anatomy.	Strong agree	54	18.8%	84	51.9%	0.004*	32.15
		Agree	102	35.4%	54	33.3%		
		Disagree	96	33.3%	21	13.0%		
		Strong disagree	36	12.5%	3	1.9%		
A3	I feel more confident in my anatomical knowledge due to cadaver dissection.	Strong agree	66	22.9%	54	33.3%	0.333	3.4
		Agree	135	46.9%	75	46.3%		
		Disagree	63	21.9%	24	14.8%		
		Strong disagree	24	8.3%	9	5.6%		
A4	Cadaver dissection helps in developing a deeper understanding of clinical correlations in anatomy.	Strong agree	72	25.0%	72	44.4%	0.021*	9.69
		Agree	153	53.1%	60	37.0%		
		Disagree	48	16.7%	18	11.1%		
		Strong disagree	15	5.2%	12	7.4%		
A5	Dissecting a cadaver improves my spatial understanding of anatomical structures.	Strong agree	54	18.8%	66	40.7%	0.003*	13.76
		Agree	162	56.3%	60	37.0%		
		Disagree	60	20.8%	24	14.8%		
		Strong disagree	12	4.2%	12	7.4%		
A6	I find cadaver dissection to be a respectful and dignified way to learn human anatomy.	Strong agree	39	13.5%	75	46.3%	0.001*	57.87
		Agree	54	18.8%	63	38.9%		
		Disagree	126	43.8%	15	9.3%		
		Strong disagree	69	24.0%	9	5.6%		
A7	I feel emotionally comfortable during cadaver dissection sessions.	Strong agree	54	18.8%	84	51.9%	0.001*	25.49
		Agree	132	45.8%	54	33.3%		
		Disagree	72	25.0%	18	11.1%		
		Strong disagree	30	10.4%	6	3.7%		
A8	Cadaver dissection should remain an integral part of medical/dental education in the future.	Strong agree	105	36.5%	84	51.9%	0.009*	11.38
		Agree	162	56.3%	54	33.3%		
		Disagree	15	5.2%	18	11.1%		
		Strong disagree	6	2.1%	6	3.7%		
A9	I feel that cadaver dissection has positively influenced my attitude toward human life and death.	Strong agree	54	18.8%	90	55.6%	0.007*	36.01
		Agree	96	33.3%	48	29.6%		
		Disagree	84	29.2%	18	11.1%		
		Strong disagree	54	18.8%	6	3.7%		
A10	The initial exposure to cadaver dissection was challenging for me.	Strong agree	102	35.4%	69	42.6%	0.652	1.64
		Agree	132	45.8%	63	38.9%		
		Disagree	36	12.5%	18	11.1%		
		Strong disagree	18	6.3%	12	7.4%		

The mean formative score for the BDS group (68.16±7.84) was lower than that for the mean formative score of the MBBS group (73.76±7.64), indicating that MBBS students performed better on formative assessments. This difference may suggest variations in learning approaches, curriculum emphasis, or assessment familiarity between the two groups (Table 3).

**Table 3:** Mean and quartile of formative score of study groups. MBBS: Bachelor of Medicine and Bachelor of Surgery, BDS: Bachelor of Dental Surgery, SD: standard deviation, CI: confidence interval

Group	N	%	Mean	SD	Minimum	Maximum	Quartile 2	95% CI for mean
BDS	162	36	68.16	7.84	45	84	67.0	66.57-69.75
MBBS	288	64	73.76	7.64	56	89	75.5	71.67-75.85

The significantly lower quartile formative scores in the BDS group than in the MBBS group (p<0.001) with a medium effect size suggested a clinically significant difference in performance between the two groups (Table 4).

**Table 4:** Intergroup comparison of quartile formative scores by independent t-test. *p-value<0.05: significant. SE: standard error

Mean difference	SE of difference	Lower limit	Upper limit	t-value	p-value	Cohen's d
-5.6	1.32	-8.2	-2.98	-4.24	0.001*	0.72

A chi-square test was performed to examine the association between the study groups and their scoring categories (low and high scores). The results showed no statistically significant association between the study groups and the scoring categories (p=0.731). This finding suggests comparable performance distributions across the two groups in this context (Table 5).

**Table 5:** Association between scorers for study groups. p-value>0.05: non-significant. MBBS: Bachelor of Medicine and Bachelor of Surgery, BDS: Bachelor of Dental Surgery

Scores	MBBS		BDS		Chi-square value	p-value
	n	%	n	%		
Low score	153	53.13	81	50.00	0.135	0.731
High Score	135	46.88	81	50.00		

**Figure 2:**
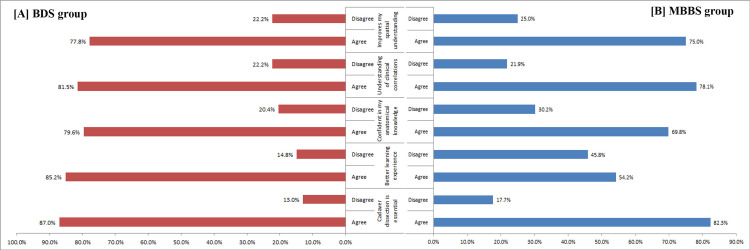
Comparison of responses between BDS (A) and MBBS (B) students regarding their perceptions of the study method in anatomy. MBBS: Bachelor of Medicine and Bachelor of Surgery, BDS: Bachelor of Dental Surgery

The MBBS students showed consistently higher agreement across all parameters. A notable number of MBBS students perceived cadaveric dissection as essential compared with BDS students. Similarly, a higher percentage of MBBS students agreed that cadaveric dissection offered a better learning experience than BDS students did. Confidence in anatomical knowledge was also rated higher by the MBBS students than by the BDS students. Both groups showed high agreement in understanding clinical correlations and spatial anatomy, with MBBS students exhibiting a slightly stronger overall perception (Figure 2).

**Figure 3:**
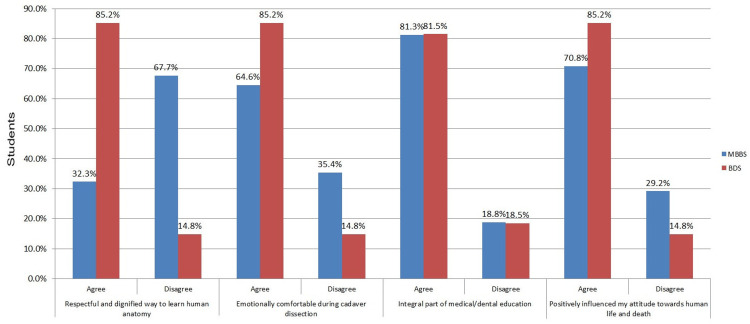
Comparison of attitude and practice level in anatomy. MBBS: Bachelor of Medicine and Bachelor of Surgery, BDS: Bachelor of Dental Surgery

The comparison of attitudes toward cadaveric dissection between MBBS and BDS students highlights notable differences. A greater proportion of MBBS students agreed that cadaveric dissection is a respectful and dignified way to learn human anatomy than BDS students. Regarding emotional comfort, a higher percentage of MBBS students expressed agreement. Both groups recognized cadaveric dissection as an integral part of their education. Finally, a higher percentage of MBBS students felt that it positively influenced their attitudes toward life and death compared to BDS students (Figure 3).

**Figure 4:**
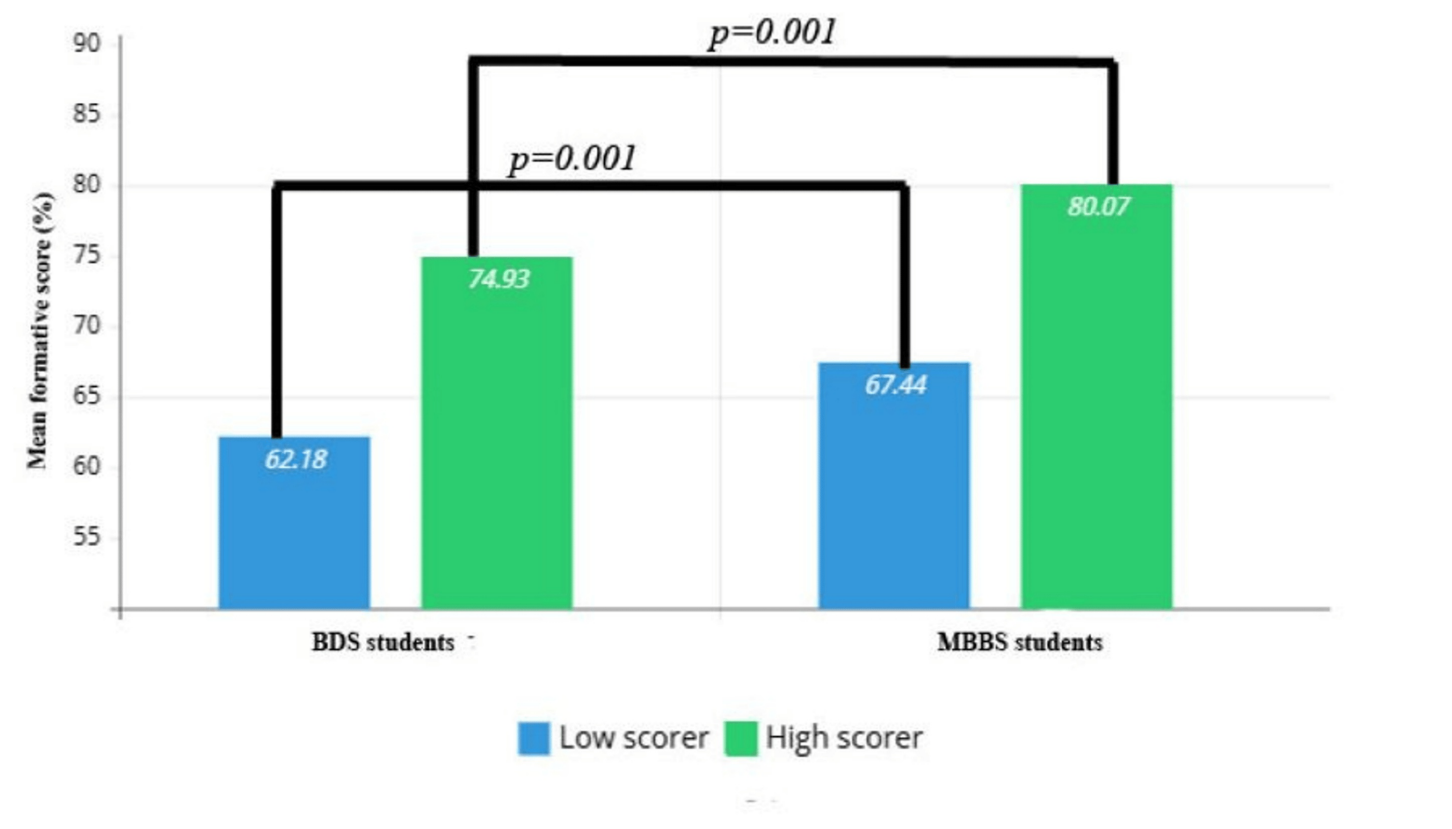
Intergroup comparison of formative scores by independent t-test.

The comparison of formative scores between low and high scorers in the MBBS and BDS students showed significant differences (p=0.001). MBBS students scored consistently higher than BDS students (Figure 4).

## Discussion

Pedagogy and evaluation of professionalism within the domain of dental or medical education are perpetually subject to scrutiny and constitute the focus of contemporary investigations that extend upon insights garnered from prior decades. It is evident that the cultivation of professional characteristics is an accumulative endeavor that necessitates targeted interventions to achieve efficacy, and one such avenue is the anatomical dissection laboratory [6]. This study presents a thorough examination of the efficacy of cadaveric dissection in teaching anatomy to first-year MBBS and BDS students. The results of our survey indicated that BDS students showed more empathy toward cadaveric dissection and believed it to be an integral part of learning. This could be due to the fact that dentistry requires high manual dexterity and precision. The cadaveric dissection might resonate more with BDS students as it aligns closely with practical skills that they will need in their future careers [9]. The significance of donors and the advantages afforded to students in their capacity to engage in cadaveric dissection should be underscored during the preliminary phases of the dissection laboratory. The significance of the time allocated to dissection should be emphasized more constructively, and students should be reassured that the opportunity to engage with a cadaver is of considerable importance, as well as being informed that they have effectively utilized a resource that will ultimately serve the public good [10,11]. Cadaveric dissection has been an important method of education to teach about the human body [1], and previous studies have been conducted to assess the perception of dental and medical first-year students toward cadaveric dissection [8,10]. However, very few studies have been conducted to compare the perception between medical and dental students.

Owing to the shortage of cadavers in recent times, virtual dissection has gained momentum as a teaching and learning modality for medical students [4,5]. In India, the conventional practice of cadaveric dissection continues to constitute the fundamental basis for the pedagogy and comprehension of anatomy, which is referred to as the “Dissection-based Anatomy Curriculum" [10]. Nonetheless, this methodology is progressively being complemented by the implementation of 3D virtual dissection within medical institutions throughout India [2]. Hisley et al. [12] noted that students taught using only the virtual dissection approach could not transfer their knowledge into the real world. According to Asad et al. [13], cadaveric dissection plays an important role in developing clinical and personal skills; however, a hybrid approach of using both cadaveric and virtual dissection methods is needed for a better understanding.

The exploration of low and high scorers further delineated the performance dynamics between the two groups. BDS students scored lower than MBBS students. Tsai and Han [14] investigated the multitude of factors that affect the adverse emotional states of dental students in the context of anatomical education and concluded that although we solicited dental students to articulate their emotional responses regarding cadavers, the sources of negative emotions may not solely stem from the direct interaction with the cadaver, but also from the perception that the gross anatomy course is superfluous for dental students. MBBS students typically cover a more extensive and detailed anatomy curriculum as their training encompasses the entire human body to prepare for a wide range of medical fields. BDS students focus primarily on the head, neck, and oral cavity, which might result in less exposure to the broader anatomical concepts tested in cadaveric dissection-based assessments. BDS students might allocate less time to anatomy than MBBS students, as their curriculum often includes other specialized subjects. BDS students may have fewer hours dedicated to cadaveric dissection or less frequent hands-on dissection experiences, leading to reduced familiarity with anatomical structures [15].

Undoubtedly, virtual dissection proves to be significantly advantageous in the realm of education and knowledge dissemination compared with cadaveric dissection; however, it ultimately fails to provide aspiring surgeons with the tactile experience of human tissue, which is an indispensable competency for future surgical practitioners. Furthermore, various anatomical variations and anomalies may occur during the examination of human anatomy. Such critical observations are likely to be overlooked by students engaged solely in anatomical studies that utilize virtual dissection. Surgical proficiency developed through the examination of human anatomy cannot be equated with simulated engagement [16].

The question of whether traditional cadaveric dissection will persist as the preferred methodology for imparting practical anatomical knowledge to medical institutions globally remains a subject of contention in this era characterized by continuously advancing innovative digital technologies. Our investigation revealed that through anatomical exploration via dissection, individuals cultivated a profound sense of empathy and reverence for the human cadaveric form. This enhanced awareness of the distinct identity of patients appears to foster a tendency among students to perceive patients as unique individuals [17]. Recognizing patients as unique individuals constitutes an essential element of medical practice, particularly given that contemporary patient dissatisfaction in the realm of medicine seldom arises from deficiencies in scientific understanding. Our investigation additionally observed that the preliminary encounter with cadavers was perceived as arduous, a conclusion corroborated by the research conducted by Surti et al. [18], which indicated an increase in anxiety levels among medical practitioners during their oral assessments, which was particularly pronounced among MBBS students. According to a study by Wilkinson et al. in 2014 [19], no significant association has been found between learning style and assessment scores. However, according to Ogut et al. [20], the learning styles affect study duration and theoretical anatomy course scores.

Clinical implications

These findings underscore the critical role of cadaveric dissection in cultivating clinical skills, empathy, and professionalism among medical and dental students. Cadaveric dissection offers tactile experience and exposure to anatomical variations. Addressing the emotional challenges associated with cadaveric dissection, especially among BDS students, can enhance their learning experiences and resilience. Emphasizing the value of cadavers as "first patients" can deepen respect for human life and improve patient care by promoting the perception of patients as unique individuals.

Limitations of the study

While the study included participants from two medical and two dental colleges, the results may not be generalizable to all medical and dental students in India or globally because of limited geographical and institutional representation. Self-reported data may introduce biases in response accuracy. The small sample size was another limitation of our study. The study's cross-sectional nature provides a snapshot of students' perceptions and attitudes at a single point in time, limiting the ability to assess changes over time or causality. Students' perceptions of cadaveric dissection may be influenced by their prior exposure, personal preferences, or biases, which were not explored in depth in the survey. Factors such as teaching quality, availability of resources, and emotional or psychological impacts of cadaveric exposure have not been thoroughly examined despite their potential influence on student perceptions and performance. The academic results derived from these evaluations cannot be exclusively ascribed to cadaveric dissection. A multitude of teaching methodologies significantly impacts these findings.

Strengths of the study

This study provides a comprehensive analysis of medical and dental students' perceptions of cadaveric dissection, incorporating diverse institutions and a large sample size. It highlights the importance of cadaveric dissection in anatomy education and its emotional and professional impact, offering valuable insights for curriculum development.

Recommendations

It would be more advantageous to undertake an examination of the head and neck region pertinent to both fields following comprehensive cadaveric dissections, supplemented by lectures or demonstration sessions that emphasize regional gross anatomy. Future research should explore longitudinal impacts of cadaveric dissection on student performance and emotional resilience. Integrating a hybrid approach combining cadaveric dissection and virtual dissection can optimize anatomy education. Additionally, addressing cadaver shortages through innovative strategies and emphasizing the ethical and professional significance of dissection can enhance student engagement and learning outcomes.

## Conclusions

In conclusion, this study demonstrated that for perception, dental students significantly favored cadaveric dissection for providing a better learning experience than alternative methods, improving spatial understanding, and aiding clinical correlation understanding. Regarding attitude, dental students more strongly associated cadaveric dissection with respect and dignity, emotional comfort, and influencing attitudes toward life and death. In practice, dental students showed greater agreement that cadaveric dissection should remain integral to education and benefit their future careers. The mean formative score for dental students was lower than the mean formative score for medical students. 
